# Four Individuals with a Homozygous Mutation in Exon 1f of the *PLEC* Gene and Associated Myasthenic Features

**DOI:** 10.3390/genes11070716

**Published:** 2020-06-27

**Authors:** Magdalena Mroczek, Hacer Durmus, Ana Töpf, Yesim Parman, Volker Straub

**Affiliations:** 1 John Walton Muscular Dystrophy Research Centre, Translational and Clinical Research Institute, Newcastle University and Newcastle Hospitals NHS Foundation Trust, Newcastle upon Tyne NE1 3BZ, UK; m.mroczek888@gmail.com (M.M.); ana.topf@ncl.ac.uk (A.T.); 2 Department of Neurology, Istanbul Faculty of Medicine, Istanbul University, Istanbul 34093, Turkey; durmushacer@yahoo.com (H.D.); parmany@istanbul.edu.tr (Y.P.)

**Keywords:** plectin, *PLEC*, myasthenia, limb-girdle muscular dystrophy

## Abstract

We identified the known c.1_9del mutation in the *PLEC* gene in four unrelated females from consanguineous families of Turkish origin. All individuals presented with slowly progressive limb-girdle weakness without any dermatological findings, and dystrophic changes observed in their muscle biopsies. Additionally, the neurological examination revealed ptosis, facial weakness, fatigability, and muscle cramps in all four cases. In two patients, repetitive nerve stimulation showed a borderline decrement and a high jitter was detected in all patients by single-fiber electromyography. Clinical improvement was observed after treatment with pyridostigmine and salbutamol was started. We further characterize the phenotype of patients with limb-girdle muscular dystrophy R17 clinically, by muscle magnetic resonance imaging (MRI) features and by describing a common 3.8 Mb haplotype in three individuals from the same geographical region. In addition, we review the neuromuscular symptoms associated with *PLEC* mutations and the role of plectin in the neuromuscular junction.

## 1. Introduction

The *PLEC* gene consists of 32 exons and is ubiquitously expressed, including in skeletal muscle, skin and heart [1]. Plectin is a large protein ranging from 4684 amino acids in the canonical form to 3447 amino acids in the smallest stable isoform. It has a unique molecular structure: the N-terminal domain is encoded by multiple short exons, whereas the central-rod domain and the C-terminal domain are encoded by single large exons. Exons 2-32 are constant, but eight different-sized forms of exon 1 exist, which undergo alternative splicing into exon 2. At least eight different plectin isoforms (1, 1a, 1b, 1c, 1d, 1e, 1f, and 1g) resulting from the different splicing of exon 1 have been identified [2]. These isoforms have various lengths, localizations, interacting partners, and specific functions. For instance, plectin 1a is localized to the outer nuclear/endoplasmic reticulum membrane system, plectin 1b to the mitochondria and plectin 1d at the Z-disks [3]. Plectin 1f, in particular in striated muscle, has been shown to interact with components of the dystrophin-glycoprotein complex [4] but also to play an important role in the neuromuscular junction (NMJ) where it interacts directly with rapsyn [5]. Plectin stabilizes the desmin cytoskeleton and interacts with its three main components: actin microfilaments, microtubules, and intermediate filaments. Other tissue specific roles depend on the specific splicing isoform and the numerous binding partners, such as α-actinin, vimentin, keratin, and desmin, which interact with different regions of the plectin protein [3,6].

Mutations in *PLEC* are associated with multiple phenotypes depending on the location of the mutation and isoform affected. Epidermolysis bullosa simplex with muscular dystrophy [EBS-MD (MIM #226670)] is caused by recessive mutations [7,8], mostly nonsense, out-of-frame insertions or deletions within exon 31 and 32, leading to premature protein termination. Individuals with EBS-MD show severe skin blistering, sometimes accompanied by skin atrophy, alopecia, and nail dystrophy, with first symptoms usually starting in infancy and even leading to premature death in childhood. The EBS-MD phenotype shows high clinical variability with a few individuals also presenting with myasthenic symptoms and additional features, such as anemia and cardiomyopathy [9]. Additionally, a phenotype of recessive limb-girdle muscular dystrophy, LGMD R17 plectin-related (MIM #613723, previously known as LGMD 2Q), was reported [10]. LGMD R17 is associated so far with only recessive truncating mutations located in exon 1f, and manifests with muscle weakness without any skin involvement. This phenotype was first described in three consanguineous Turkish families carrying a c.1_9del mutation and presenting with young adult/childhood onset muscle weakness and dystrophic changes in the muscle biopsy, without skin involvement or myasthenic features [10].

The association of plectin deficiency with muscle weakness is already well characterized. However, the role of plectin deficiency in patients with features of a congenital myasthenic syndrome is still not well understood and is only based on a few case reports. Herein, we describe four individuals with LGMD, easy fatigability, ptosis, and clinical responsiveness to acetylcholinesterase inhibitors and salbutamol, features commonly associated with myasthenic syndromes. They all carry the known c.1_9del *PLEC* mutation in homozygosity. All four patients included in this study originate from the same region near the Black Sea, in Turkey. We also identified a shared haplotype in three individuals suggesting a common origin of the mutation. Patients with myasthenic symptoms of unknown origin should be tested for mutations in the *PLEC* gene.

## 2. Materials and Methods

### 2.1. Clinical Assessment

Four patients with LGMD from unrelated families born to consanguineous parents were evaluated at the Department of Neurology, Istanbul Faculty of Medicine, Istanbul University, Turkey. The efficacy of oral salbutamol administration was assessed by muscle strength measurements, the 6-min walk test (6MWT) and spirometry (forced vital capacity; FVC). Muscle magnetic resonance imaging (MRI) was performed on a 1.5-Tesla MR scanner (1.5 T Philips Achieva, Philips Medical Systems, Best, Netherlands), using conventional T1-weighted and T2-weighted SPIR (Spectral Presaturation with Inversion Recovery) axial images of the thigh. Eight-micrometer sections from shock-frozen tissue samples were stained with hematoxylin and eosin, modified Gomori trichrome, acid phosphatase, periodic acid–Schiff, NADH dehydrogenase, succinate dehydrogenase, cytochrome *c* oxidase, and oil red O using standard procedures.

### 2.2. Standard Protocol Approvals, Registrations, and Patient Consents

Photographs and blood samples from patients and family members were obtained upon written informed consent according to the Declaration of Helsinki. The study protocol was approved by the Istanbul University Institutional Review Board for Research with Human Participants. DNA samples were submitted to the Newcastle Medical Research Council (MRC) Center Biobank for Neuromuscular Diseases for which ethical approval was granted by the NRES (National Research Ethics Service) Committee North-East—Newcastle & North Tyneside 1 (reference 08/H0906/28). Informed written consent was given by the patients. Phenotype data collection was performed using the PhenoTips online software tool (https://phenotips.com).

### 2.3. Genetic Analysis

All families characterized in this study were consanguineous and showed a recessive inheritance pattern. Three individuals (family A, B and C) were included in the MYO–SEQ project [11]. For these patients, whole exome sequencing (WES) was performed at the Broad Institute’s Genomics Platform, using Illumina exome capture on a cohort of >1800 patients with limb-girdle muscle weakness. Exome data was analyzed using standard filtering criteria (i.e., VEP moderate to high and MAF <1% and a gene list of 429 genes associated with muscle disease). The patient from family D was diagnosed by exome sequencing analysis performed at the Intergen Genetic Laboratory in Ankara, Turkey and the *PLEC* mutation was confirmed by Sanger sequencing. The size of the haplotype for three patients (A, B and C) was estimated using unfiltered WES data. Annotation is based on gene accession number NM_201378, ENST00000356346 and NP_958780.1.

## 3. Results

### 3.1. Clinical Presentation

**Figure 1:**
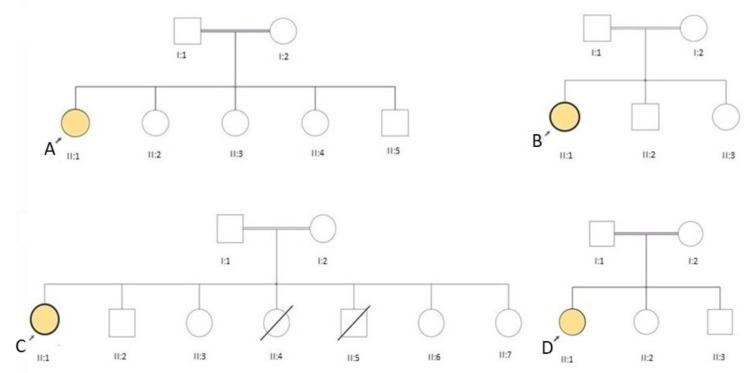
Pedigrees of our four LGMD R17 patients carrying the c.1_9del mutation in homozygosis. Patients A, B, C and D are indicated.

**Figure 2:**
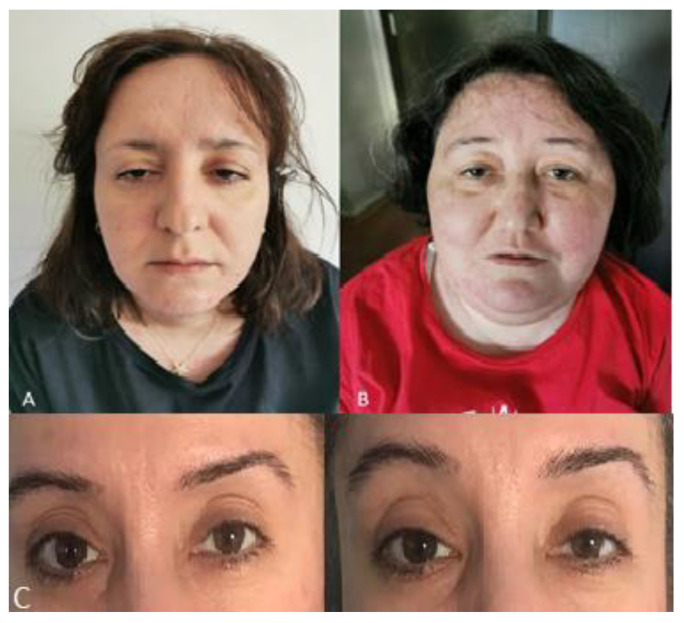
(**A**) Patient B showing ptosis and facial weakness at age 39 years old; (**B**) Patient A showing ptosis and facial weakness at age 38 years old; (**C**) Patient D with fatigable ptosis.

All four female patients were born to consanguineous parents (Figure 1), but with no family history of a neuromuscular disorder. All presented with slowly progressive proximal muscle weakness, easy fatigability, and muscle cramps. Two of them (C and D) had delayed motor milestones; three were slower than their peers during childhood, only patient B presented at 26 years old. Their neurological examination revealed fatigable ptosis (Figure 2), nasal speech, tongue weakness, neck flexor weakness, proximal weakness and paraspinal muscle weakness (Table 1). A high arched palate, mild scoliosis, and bilateral pseudohypertrophy in the gastrocnemius muscle were noted in all. Two patients (A and C) were wheelchair-bound at age 35 and 39 years. Serum creatine kinase (CK) levels were elevated (Table 1) and electromyography (EMG) showed myopathic changes. Patients A and B were negative for anti-AChR and anti-MUSK antibodies. In retrospective, the myasthenic symptoms, such as mild ptosis, were present since childhood, in patients A, C, D, and since her early twenties in patient B. All patients showed easy fatigability since childhood.

**Figure 3:**
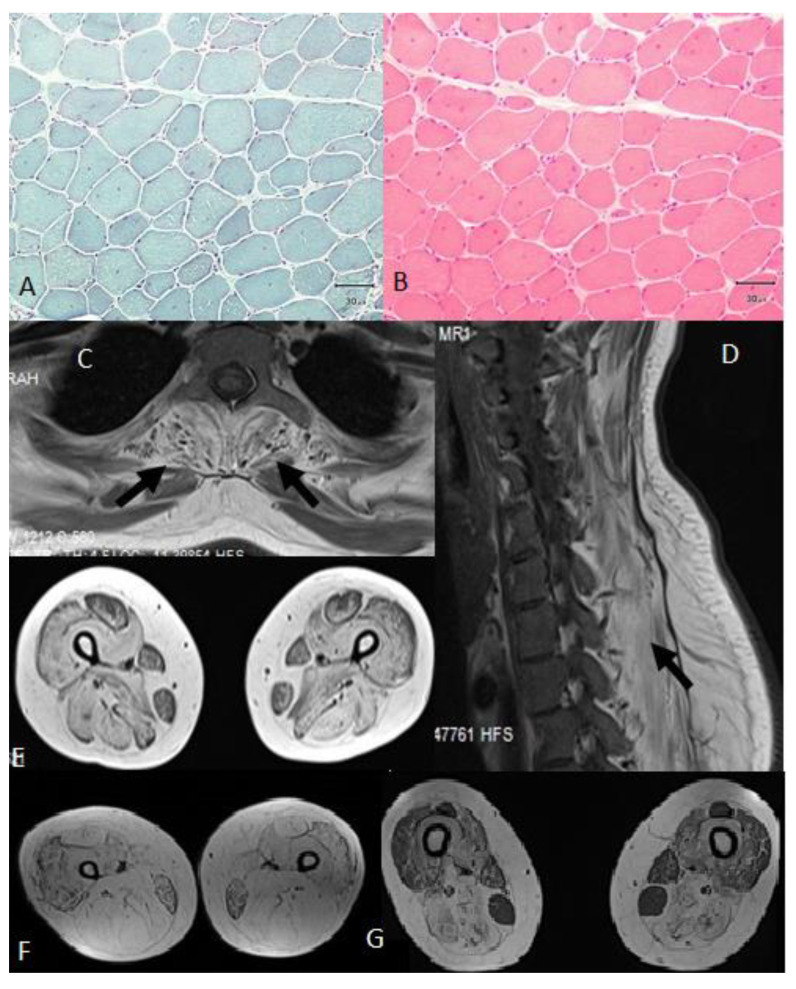
(**A**) and (**B**) A muscle biopsy from the deltoid muscle of patient D showed myopathic changes with increased numbers of internal nuclei (over 10%) and with a few angular atrophic fibers; (**C**), (**D**) and (**E**) A muscle MRI of patient D showed paraspinal muscle involvement and good preservation of the sartorius and gracilis muscles in the lower limbs. (**F**) The muscle MRI of patient A showed advanced fatty transformation, but fairly good preservation of the gracilis muscles. (**G**) The muscle MRI of patient B showed clear pathology of the adductor magnus, adductor longus, biceps femoris, semitendinosus, and semimembranosus muscles and sparing of the gracilis muscles with good preservation of the rectus femoris and sartorius muscles.

All patients had high jitter in single-fiber EMG. Two patients who had milder weakness had over 10% decremental response in repetitive nerve stimulation (RNS). Muscle biopsy analysis showed dystrophic changes in three patients and myopathic changes in one, with increased number of internal nuclei (over 10%) and a few angular atrophic fibers in all (Figure 3A,B). Muscle MRI showed relative sparing of the rectus femoris, gracilis and sartorius muscles in three patients (Figure 3C–G). Cardiac examinations including echocardiogram (ECHO) and 24 h Holter monitoring were normal. Treatment with pyridostigmine 180 mg/day and salbutamol 8 mg/day showed benefit in all patients. Salbutamol was well tolerated with no noted side effects. After six months of therapy, patient A was functionally better and noticed that she could use her arms for much longer without fatigue. Her baseline and 6-month scores according to the MRC scale were similar. Baseline respiratory function tests of patient B revealed a vital capacity (VC) of 53% and an forced vital capacity (FVC) of 54%. After 6 months of salbutamol treatment, her FVC increased to 66%. She also performed better in the 6MWT, with an increase from 274 m to 326 m. She only needed unilateral support for walking. Patient C’s baseline and 3-month MRC scores were similar, but her FVC in sitting increased from 57% to 62%. She was able to use her arms and hands more effectively and felt less tired after six months of salbutamol treatment. Salbutamol was well tolerated except for mild tremor in the hands. Patient D started treatment only recently and already reported that she felt less fatigued and was able to climb stairs more easily. Her 6MWT rose from 150 m before treatment to 180 m after treatment, while her VC increased respectively from 59% to 74%.

### 3.2. Variant Characterization

Whole exome sequencing identified a homozygous *PLEC* variant (hg19: chr8:145047630_1450476308delGCCGGCCAT; c.1_9delATGGCCGGC; p.[Met1_Gly3del]) in four unrelated patients; its segregation with the disease was ascertained by Sanger sequencing and revealed a heterozygous variant in a healthy sister and the unaffected parents. This variant is absent in the control population (https://gnomad.broadinstitute.org/region/8-145047531-145047731) and has already been associated with LGMD R17 in three consanguineous families of Turkish origin [10]. No other likely candidate gene was identified in the targeted analysis of the exomes; in particular no disease-causing variant in any of the known congenital myasthenic syndrome (CMS) genes (*RAPSN, CHRNE, AGRN, ALG2, CHAT, CHRNA1, CHRNB1, CHRND, CHRNE, COLQ, DOK7, GFPT1, GMPPB, MUSK, PREPL* and *SLC5A7*) was found.

**Figure 4:**
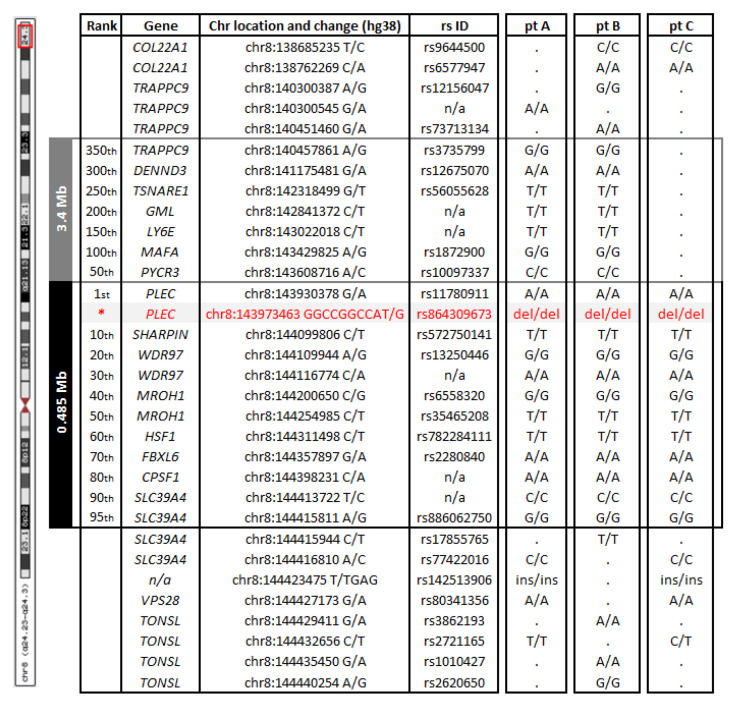
Common haplotype based on WES data. Patients A, B, and C shared a 0.485 Mb haplotype (defined by 95 variants and encompassing 17 genes; shown in black) from chr8:143930378 to chr8:144415811. Patients A and B shared a larger haplotype (3.4 Mb, in grey), defined by ~350 variants from chr8:140457861 to chr8:143608716 and encompassing 45 genes. Selected variants and their genotypes are indicated both within and outside the haplotype. A dot indicates that the variant was not present in that patient. The *PLEC* mutation identified in the four Turkish patients is shown in red.

In addition, based on the WES data for three of the patients (A, B and C), a shared 0.485 Mb (hg38:chr8:143930378-144415811) homozygous haplotype defined by 95 single nucleotide and small in/del variants, and surrounding the *PLEC* mutation was identified, suggesting a founder event for this variant. A larger shared haplotype of 3.4 Mb (hg38:chr8:140457861-143608716) defined by an additional ~350 variants can be seen for only two of the patients (A and B), indicating that a recombination event took place in patient C (Figure 4). The 0.485 Mb and the larger 3.4 Mb regions encompass 17 and 45 genes, respectively. According to the medical history, the families were not related (previous 4–5 generations were analyzed); however, their ancestors all came from Giresun, a city by the Black Sea in Turkey.

## 4. Discussion

We present four patients from unrelated families carrying the same *PLEC* mutation and presenting with limb-girdle weakness and myasthenic symptoms. All four patients had shoulder and pelvic girdle weakness with variable degree of fatigable ptosis, nasal speech, and swallowing difficulties. Paraspinal muscle weakness was also very prominent in our patients. All complained of easy fatigability and muscle cramps. The age of onset of symptoms varied from the infantile period with delayed motor milestones to early adulthood. They all had elevated serum CK levels and a high jitter on RNS. All patients benefited from pyridostigmine and salbutamol treatment.

The most common neuromuscular phenotype associated with mutations in *PLEC* is epidermolysis bullosa simplex with muscular dystrophy (EBS-MD; MIM #226670). Symptoms include skin blistering and ocular and neuromuscular involvement later in life [9]. The disease is inherited in an autosomal recessive manner and is associated with a dissociation of desmin filaments and, consequently, protein aggregates positive for desmin, syncoilin, and synemin accumulate in muscle fibers. Myofibrillar changes can be observed histologically and secondary mitochondrial pathology has been described [12]. Additionally, some patients present with systemic involvement (e.g., gastroenterological, dental, urogenital and cardiac) [13,14,15]. A few individuals with EBS-MD show myasthenic features, such as fatigability, ptosis, dysphagia or nasal speech [7,16,17]. However, these features were not investigated systematically and additional tests, such as SF-EMG had previously only been performed in a single case. The data from our four patients showed that the myasthenic phenotype may be more common in patients with *PLEC* mutations but may be overlooked when the clinical presentation is only mild. All EBS-MD mutations identified so far, apart from one, were in exons common to all plectin isoforms, and in the majority of cases lead to premature termination of protein translation. Other phenotypes associated with *PLEC* mutations are autosomal recessively inherited EBS with pyloric atresia (EBS-PA; MIM #612138) [18] and autosomal dominantly inherited EB simplex Ogna type (EBS-OG; MIM #131950) [19]. These diseases are associated mainly with C-domain *PLEC* mutations.

In addition, patients with recessive *PLEC* mutations and a LGMD phenotype, but without any skin involvement (LGMD R17, previously known as LGMD 2Q), have also been reported. The phenotype was first described in three consanguineous Turkish families (six affected individuals in total) carrying the same homozygous c.1_9del mutation identified in the patients described here. This 9 bp deletion contains the ATG initiation codon located in exon 1f, and results in impaired translation of the plectin 1f isoform exclusively [10]. More recently, another truncating variant in exon 1f (p.Glu20Ter), also resulting in a LGMD R17 phenotype, has been reported [20].

The four families homozygous for the c.1_9del mutation that are characterized in this manuscript show many common features with the previously described families with LGMD R17, and none of the patients showed skin or nail involvement. However, there were some important differences between the patients described in this manuscript and those reported by Gundesli et al. [10] (Table 1). All patients displayed clear myasthenic symptoms, such as ptosis, fatigability, nasal speech, and a positive clinical response to the treatment with pyridostigmine and salbutamol. Additionally, one patient’s onset was in adulthood with a completely asymptomatic childhood period. All four patients described in this manuscript had paraspinal muscle weakness and calf hypertrophy. It is possible, however, that the myasthenic features might indeed not have been manifested in patients that were even younger than the ones we report. Alternatively, the more severe muscular dystrophy phenotype seen in the c.1_9del patients reported by Gundesli et al. might have masked the myasthenic symptoms.

Our patients come from unrelated consanguineous families from the Black Sea region in Turkey, as were the individuals described by Gundesli et al. who identified –using polymorphic DNA markers—a shared haplotype of ~2.3 Mb upstream of the *PLEC* variant in five of their patients [10]. Our haplotype analysis using WES data for three of our patients confirms the founder effect for the *PLEC* variant in the Turkish population but also delineates the breakpoints and size of the haplotype more clearly at 3.8 Mb.

**Figure 5:**
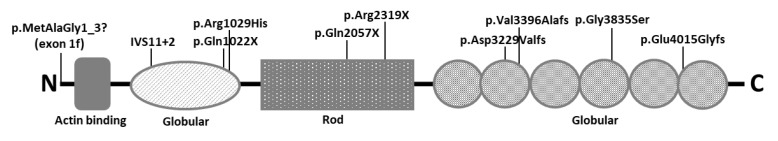
Location of *PLEC* mutations associated with myasthenic phenotypes. Most cases are compound heterozygous for null mutations located in the globular domains; the only homozygous mutation with myasthenic phenotype described so far, and in this article, is in exon 1f [21,22,23,24].

Up until now, myasthenic phenotypes were frequently associated with mutations in the C-domain of *PLEC.* However, the cases described here carry the c.1_9del mutation, located in exon 1 of the plectin 1f isoform, and no other rare damaging variants in the *PLEC* actin binding domain have been detected (Figure 5). Plectin 1f plays an important role in the NMJ and interacts directly with rapsyn [5]. Our findings support the hypothesis of a crucial role of plectin 1f isoform in the NMJ and in the pathogenesis of myasthenic features. Although we only found a decremental response of 10% or more on RNS in two of our patients, the other two patients had severe weakness due to the muscular dystrophy, which might have masked the dysfunction of the NMJ. The patients who had less severe weakness and more prominent decremental response on RNS, had the most benefit from treatment. Early diagnosis and treatment of these patients may be more effective and lead to an improved quality of life.

To the best of our knowledge, only a few cases of EBS-MD with myasthenia have been described so far (Table 2). However, myasthenic features in EBS-MD patients may be missed if they are not investigated systematically and no additional tests such as SF-EMG are performed.

Plectin is a structural scaffolding protein concentrated at sites of mechanical stress and in the Z-disc. In *PLEC* patients with myasthenic features, neuromuscular junctions showed abnormal morphology with disrupted structures and degeneration [5,26]. At the molecular level, it was shown that plectin 1f plays an important role in the stability of the NMJ [4]. Electron microscopy analysis of muscle biopsies of patients with start codon loss in 1f isoform showed empty spaces between the sarcolemma and the contractile elements [10]. It was suggested that PLEC 1f interacts with desmin intermediate filaments (IFs) in the postsynaptic domain of the NMJ and plays a central role in their stability by directly interacting with rapsyn. Staining performed specifically for the PLEC 1f isoform in the NMJ showed co-localization with acetylcholine receptors (AChRs) [5] and in plectin-deficient myoblasts, AChRs are highly mobile and cannot form stable clusters. PLEC 1f also interacts with desmin and studies in PLEC 1f deficient myoblasts showed that without such a linkage the structural network of NMJ collapses [5,17]. Conditional plectin knockout mice (Pax7-Cre/cKO) with postsynaptic absence of plectin, resulted in a phenotype similar to EBS-MD-MyS, with severe lack of coordination, reduced motility, and shortened life span. In this model, uncoupling of AChRs from IFs resulted in severe disorganization and fragmentation of the NMJ and depletion of AChRs. Instead, presynaptic lack of plectin did not cause reduced muscle strength in mice lacking P1c plectin isoform [5].

Phenotypes associated with *PLEC* mutations are highly variable, which may reflect the multiple binding partners of the plectin protein. The cause of this clinical variability is not yet fully understood but could be explained by the location of the mutations in different domains and various isoforms [27]. As many patients with plectin deficiency have not been thoroughly investigated for NMJ defects, it may be that myasthenic features have been underestimated. Our cases carrying the c.1_9del *PLEC* mutation broaden the LGMD R17 phenotype with onset in adulthood along with extraocular, facial, and tongue involvement. Individuals with *PLEC* mutations should be carefully examined for myasthenic features and SF-EMG is crucial to confirm the diagnosis.

## Figures and Tables

**Table 1:** Comparison of clinical phenotypes between patients with c.1_9del *PLEC* deletion.

ID	Age (Years)	Age of Onset (Years)	Motor Milestones	First Symptom	Fatigability	Weakness (MRC)	Calf Pseudohypertrophy	Wheelchair (Age)	CK (*N* < 220 U/L)	EMG	Biopsy	Refs
A	37	6	Normal	Difficulty in walking	Yes	Ptosis, neck flexor 2/5, upper proximal (3/5), distal (4/5), lower proximal (2/5), distal (4/5), paraspinal and abdominal muscle weakness	Yes	Yes (38)	1379	Myopathic, high jitter in SF-EMG	Dystrophic, increased number of internal nuclei, a few angular atrophic fibers	This study
B	38	26	Normal	Difficulty in climbing stairs	Yes	Bilateral ptosis, neck flexors 3/5, upper proximal (4/5), upper distal 4/5 lower proximal (3/5) and distal (4/5), paraspinal and abdominal muscle weakness	Yes	No	3290	Myopathic and high jitter in SF-EMG. 10% decremental response in Trapezius muscle on RNS	Dystrophic increased number of internal nuclei, a few angular atrophic fibers	This study
C	40	Early childhood	Delayed—Age of onset for walking 2.5 years	Delayed walking, slower than her peers	Yes	Mild ptosis, nasal speech, tongue weakness, upper proximal 2/5, distal 3/5; lower proximal 1-2/5, distal 2/5, paraspinal and abdominal muscle weakness	Yes	Yes (35)	4980	Myopathic, spontaneous pathogenic activity, high jitter in SF-EMG. 8.7% decremental response in trapezius on RNS	Dystrophic, increased number of internal nuclei, a few angular atrophic fibers	This study
D	27	Early childhood	Delayed	Difficulty in climbing up stairs	Yes	Bilateral mild ptosis, upper proximal (4/5), distal (5/5), lower proximal (3/5), distal (4/5), paraspinal, and abdominal muscle weakness	Mild	No	6200	Myopathic, spontaneous pathogenic activity, high jitter in SF-EMG. 12% decremental response in trapezius on RNS	Dystrophic, increased number of internal nuclei, a few angular atrophic fibers	This study
VI:1 (*)	19	Early childhood	Delayed walking at 3 years	Delayed milestones, difficulty climbing stairs	No	No facial involvement, proximal muscle strength of 3+/5	No	No	5584	Myopathic	Dystrophic	Gundesli et al. [10]
Family II patient 20	4	2	Normal	Muscular weakness	No	NI	No	No	4159	NI	Dystrophic	Gundesli et al. [10]
Family III patient 47	5	Early childhood	Delayed walking at 2.5 years	Difficulty climbing stairs, slower in running than peers	No	NI	No	No	3704	NI	Dystrophic	Gundesli et al. [10]

EMG: Electromyography; SF-EMG: single-fiber electromyography; RNS: repetitive nerve stimulation; NI: No information. (*) Three additional similar individuals in the family.

**Table 2:** Review summary of cases with EBS-MD-MyS or EBS-MD with myasthenic features.

Type	Mutation	Consang.	Clinical Features	Onset	RNS/SF-EMG	Response to Treatment	Refs.
EBS-MD-MyS	c.6169 C>T; p.Gln2057X c.12043dupG; p.Glu4015GlyfsX69	no	AW, B, EBS, EO, F, FW, LW, P, PW, WD	Childhood	RNS 11–25% decrement	Positive to 3,4-DAP, negative to AchE inhibitors	[21]
EBS-MD-MyS	c.6955 C>T; p.Arg2319X c.12043dupG; p.Glu4015GlyfsX69	no	AW, B, EBS, EO, F, FW, LW, P, R, W, WD	Early Childhood	RNS 67% decrement	Negative	[21]
EBS-MD-MyS	c.3064 C>T; p.Gln1022X c.11503G>A; Gly3835Ser	no	AW, B, F, FD, FW, LW, P, PW	Childhood	SF-EMG negative	Negative	[22]
EBS-MD-MyS	c.3086 G>A; p.Arg1029His c.9679_9766del88; p.Asp3229ValfsX21	no	AW, BW, DMM, EBS, F, FW, LW, P, PW, RFM	Congenital	RNS 12% decrement, SF-EMG positive ^1^	Positive to pyridostigmine	[23]
EBS-MD-Mys	c.10187_10190delTGTC, p.Val3396AlafsX11 IVS11+2 T>G	no	AW, DMM, EBS, EO, F, FD, FW, H, IGH, LW, P, PW, RFM, W	Congenital	RNS 16% decrement	Positive to pyridostygmine	[24]
EBS-MD with ptosis	?	no	AW, DMM, EBS, FW, LW, P, PW, R, WD	Congenital	?	?	[16]
EBS-MD with congenital myasthenia gravis	?	yes	EBS, F; congenital myasthenic syndrome treated with thymectomy	Congenital	?	?	[8]
EBS-MD with ocular signs	?	no	EBS, LW, PW, WD, slightly restricted eye movement, and reduced strength in eyelid closure	Congenital	?	?	[25]

AW: axial weakness; B: bulbar weakness; DMM: delayed motor milestones; EBS: epidermolysis bullosa simples; EO: external ophthalmoplegia; F: fatigue/exercise intolerance; FD: feeding difficulties/dysphagia; FW: facial weakness; H: hypotonia; IGH: intrauterine growth retardation; LW: limb weakness; P: ptosis; PW: proximal weakness; R: respiratory weakness; RFM: reduced fetal movements; RNS: repetitive nerve stimulation; SC: scoliosis; A: alopecia; SF-EMG: single-fiber electromyography; W: wheelchair-bound; WD: walking or running difficulties; 3,4-DAP: 3,4-diaminopyridine; ?: unknown. Variant annotation is based on NM_201378, ENST00000356346 and NP_958780.1. ^1^ apparent single-fiber action potentials showed increased jitter and blocking.
